# A Solar Position Sensor Based on Image Vision

**DOI:** 10.3390/s17081742

**Published:** 2017-07-29

**Authors:** Adolfo Ruelas, Nicolás Velázquez, Carlos Villa-Angulo, Alexis Acuña, Pedro Rosales, José Suastegui

**Affiliations:** 1 Facultad de Ingeniería, Universidad Autónoma de Baja California, Blvd. Benito Juárez S/N, Mexicali 21280, Mexico; alexis.acuna@uabc.edu.mx (A.A.); prosales@uabc.edu.mx (P.R.); asuastegui@uabc.edu.mx (J.S.); 2 Instituto de Ingeniería, Universidad Autónoma de Baja California, Calle de la Normal S/N, Mexicali 21280, Mexico; nicolas.velazquez@uabc.edu.mx (N.V.); villac@uabc.edu.mx (C.V.-A.)

**Keywords:** tracking, vision, solar position

## Abstract

Solar collector technologies operate with better performance when the Sun beam direction is normal to the capturing surface, and for that to happen despite the relative movement of the Sun, solar tracking systems are used, therefore, there are rules and standards that need minimum accuracy for these tracking systems to be used in solar collectors’ evaluation. Obtaining accuracy is not an easy job, hence in this document the design, construction and characterization of a sensor based on a visual system that finds the relative azimuth error and height of the solar surface of interest, is presented. With these characteristics, the sensor can be used as a reference in control systems and their evaluation. The proposed sensor is based on a microcontroller with a real-time clock, inertial measurement sensors, geolocation and a vision sensor, that obtains the angle of incidence from the sunrays’ direction as well as the tilt and sensor position. The sensor’s characterization proved how a measurement of a focus error or a Sun position can be made, with an accuracy of 0.0426° and an uncertainty of 0.986%, which can be modified to reach an accuracy under 0.01°. The validation of this sensor was determined showing the focus error on one of the best commercial solar tracking systems, a Kipp & Zonen SOLYS 2. To conclude, the solar tracking sensor based on a vision system meets the Sun detection requirements and components that meet the accuracy conditions to be used in solar tracking systems and their evaluation or, as a tracking and orientation tool, on photovoltaic installations and solar collectors.

## 1. Introduction

The increase in fossil fuels and electric energy generation costs has driven the development of technologies that take advantage on renewable energy sources. Solar energy is one of them—it is abundant and can be used in diverse applications, for instance water heaters, solar kitchens, process heat generation in industries, and electric energy generation.

Solar capture technologies have better performance when the direction of the Sun’s rays is normal to the surface of the capture, however, this is not always possible due to the continuous apparent movement of the Sun. To deal with this, there has been a great variety of developments in solar tracking systems [1,2,3], where it has been found that in photovoltaic systems with tracking on one or two axes, it is possible to capture 30 to 40% more energy in comparison with a fixed one [4,5,6,7]. When talking about concentrating solar power with solar collectors, the tracking system needs to be precise, for the Sun rays to fall upon the collector’s acceptance angle to ensure a satisfactory thermal behavior [8,9].

In the literature, different algorithms for control and automation of solar tracking have been reported; they can be classified into open-loop, closed-loop and hybrid control systems. The first one uses astronomic equations to determine the location of the Sun and position the collection surface area [10,11,12,13]. For closed-loop systems, differential light sensors based on phototransistors [14,15,16], photo resistors [9,17,18] photovoltaic cells [19,20] and image processing based systems [21,22,23,24,25] are used. For the third strategy, a hybrid is used, on which the Sun´s position is calculated to locate it to a nearby focus point, and from then on, the sensor itself makes a precise adjustment [26,27,28,29].

On papers about tracking systems, their design, construction, development and functionality tests are regularly mentioned. Despite being the main objective, when different control systems are being tested to determine if they meet the policies and standards to be used to test solar tracking systems, a specific process that can determine the solar focus error, or the fact that the tracking system evaluation doesn’t depend on the capture system which being used on, hasn’t been reported.

Therefore, it has become necessary to develop a sensor to evaluate the accuracy of a solar tracking system, regardless of its application. Inspired on the visual systems with image processing [23,24,25], that have been proven useful to determine the focus error on pixels, this document describes the design, construction, characterization and evaluation of a sensor based on vision to determine the focus error of a surface or solar collector, with the objective of evaluating solar tracking systems or its use as a sensor for solar tracking systems, not limiting its use to any particular orientation or level surfaces in its installation. The sensor is designed to be used on any surface such as radiometric stations (for example the SOLYS 2 used to validate the sensor), parabolic trough collectors, parabolic dishes and photovoltaic panels, the robust sensor can withstand the severe weather conditions under which solar collectors already work. 

## 2. Materials and Methods

**Figure 1:**
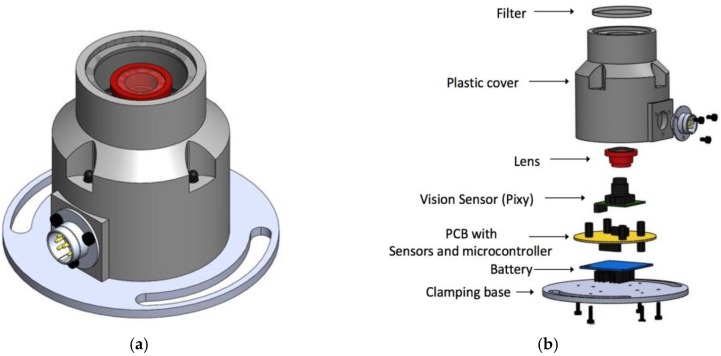
Mechanical design and geometry of the solar position sensor: (**a**) View of the assembled sensor; (**b**) Exploded view of sensor.

The solar position sensor has a cylindrical geometry, as seen in Figure 1a, where one can find the assembly structure and the electronic devices that make up the sensor Figure 1b shows the upper tempered darkened glass with a thickness of 3 mm, which acts as the solar radiation filter with ultraviolet and infrared wavelengths, the wave type that is not visible to the sensor that can cause heating and deterioration of the vision sensor. The filter is made from a Lens Protection lens of shade 14 used in welding helmets. This lens blocks artificial light or diffuse solar radiation, so that the detection of the Sun is not affected. The structure has connectors and a holding base with neodymium magnets and grips to hold it, occasionally or permanently, to the base to be evaluated.

**Figure 2:**
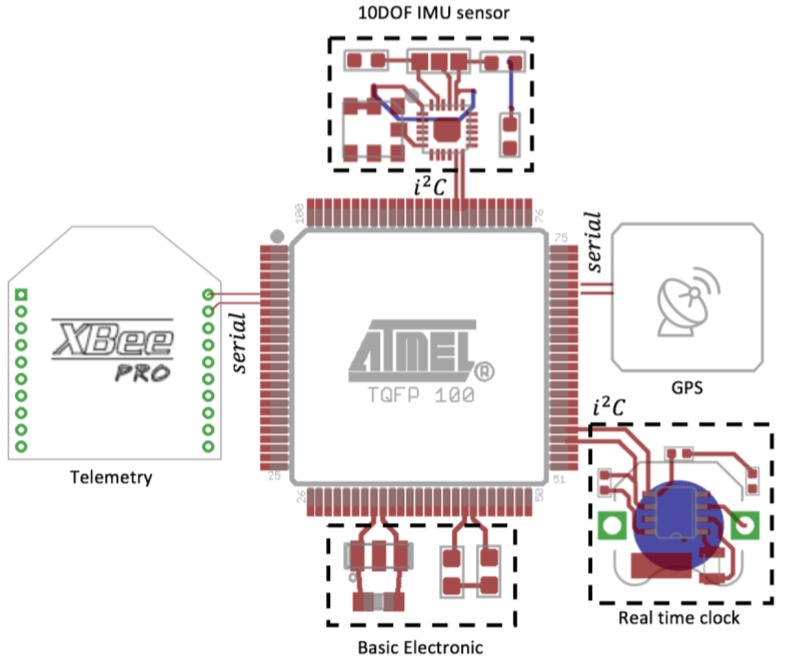
Block diagram of the hardware architecture of the solar position sensor.

The electronic circuit of the solar position sensor (see Figure 2) consists of the 8-bit Atmega2560 microcontroller with 256 KB of flash memory and a working voltage of 5 V at a temperature ranging from −10° to 85° centigrade. For this to work, basic electronics are added, consisting of a 16 MHz oscillator, 5 V regulator, a capacitor network to eliminate electrical noise and a voltage leveling stage from 5 to 3.3 V for the pressure and inertial sensor. The inclination and orientation are obtained with the mpu9250 9-axis inertial measurement sensor, which contains an accelerometer, gyroscope and magnetometer internally with working temperatures of −40° to 85° centigrade at 3.3 V. Pressure, temperature and humidity readings are obtained with the integrated BME280, which can work from −40° to 85° centigrade at 3.3 V.

The position of the Sun is obtained by image processing through the Pixy vision sensor (CMUcam5) based on the ARM-CortexNXPLPC4330 processor with working temperatures from −40° to 85° centigrade at 5 V. This sensor has the ability to detect colors with a 320 × 200 pixels resolution. 

**Figure 3:**
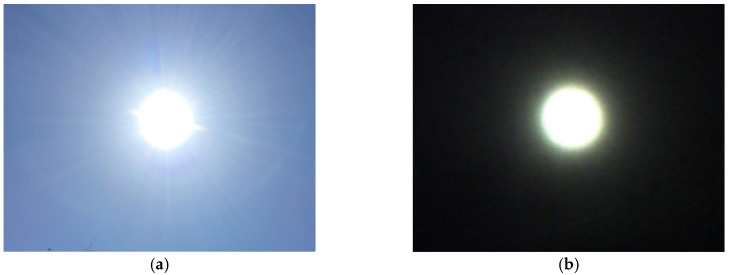
Image of the Sun viewed from the sensor (**a**) Real view of the Sun; (**b**) Sun viewed through the filtered visual sensor.

**Figure 4:**
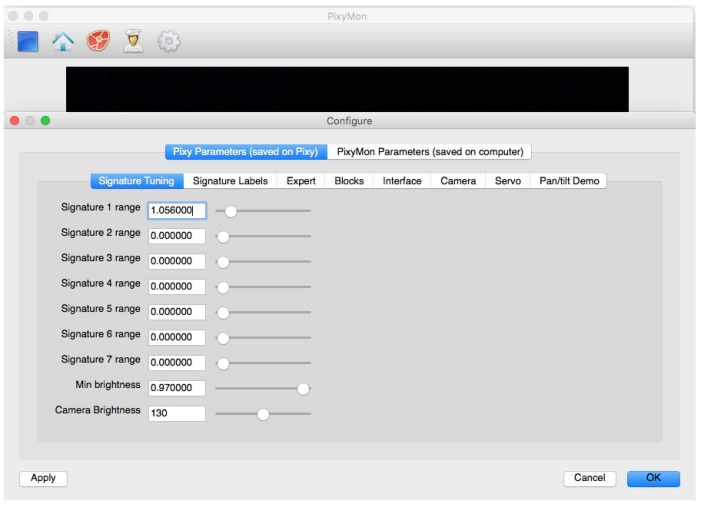
Interface and parameters of the vision sensor in the Pixymon environment.

The detection strategy is based on capturing the image of the Sun through the filter. In Figure 3a, we can see the real image of the Sun, while in Figure 3b, from the vision sensor through the filter, it is observed that the light of the Sun is identified as a clear object, which is why the Pixy sensor is configured independently through the Pixymon software with the parameters of Figure 4, with which it is able to identify the object and send its position in pixels by the i2c protocol towards the microcontroller.

**Figure 5:**
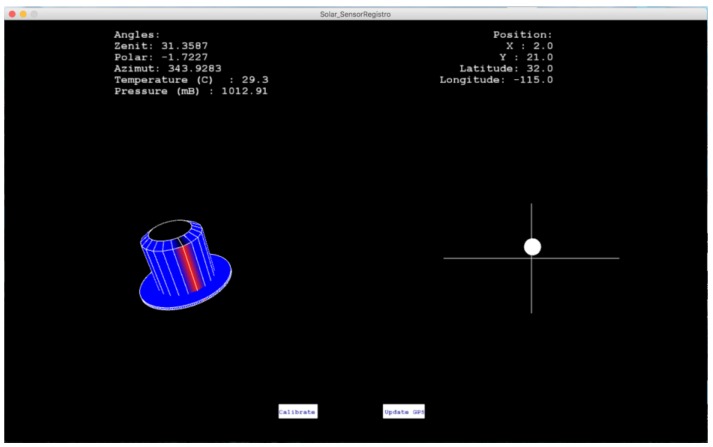
User interface of the solar position sensor.

The acquisition of data or telemetry is done through serial communication and the Xbee Pro S1 device. The microcontroller sends and receives data through the serial port and transmits it through the previously mentioned module. The receiver computing equipment contains a Java user interface made with the help of the Processing 3.0 platform. Figure 5 presents a screenshot showing the inclination, orientation, temperature, pressure, humidity, latitude, longitude and the focus error of the Sun. The information is displayed in real time and at the same time is stored in a text file. In addition it contains options to calibrate the magnetometer and adjust the time from the GPS.

**Figure 6:**
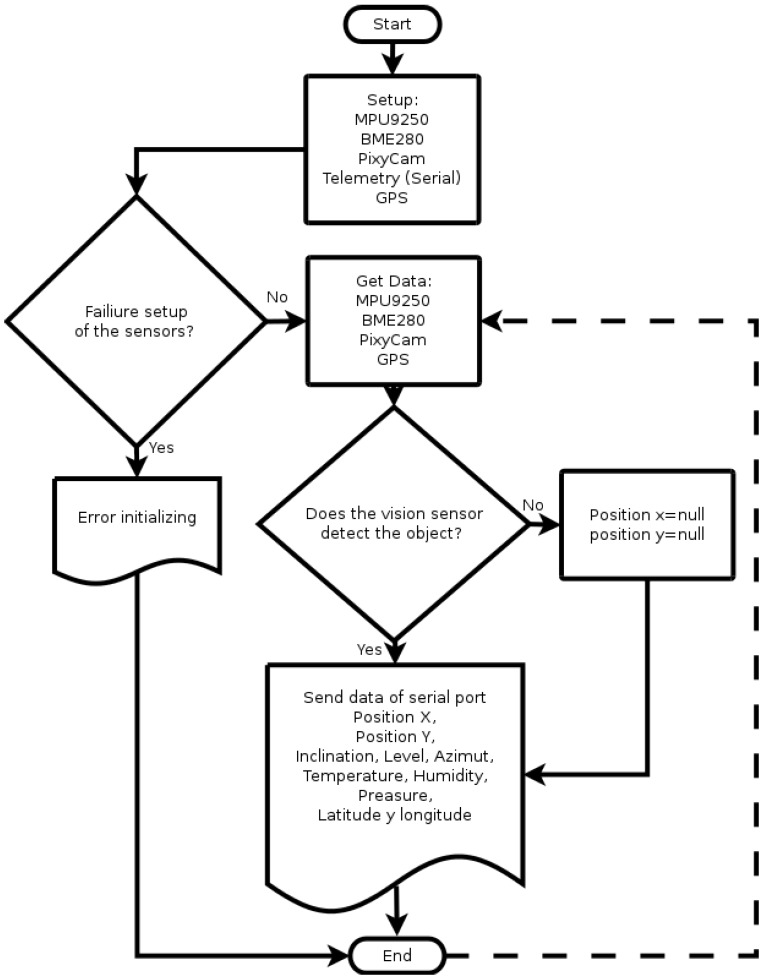
Flow diagram implemented in the ATmega microcontroller.

The algorithm to determine the focus error of Figure 6, which was implemented in the ATmega microcontroller, starts by loading the calibration values and initializing the inertial measurement sensor (MPU9250), temperature, humidity and altitude (BME280), GPS, telemetry and the Pixy vision sensor. If any of the sensors fail at initialization, an error message is sent to the interface and the cycle is terminated. When the sensors are initialized correctly, the positions of the vision, inclination, level, azimuth, temperature, humidity, latitude and longitude sensors are obtained. In the case that the vision sensor does not detect an object, null values are assigned to the positions in order for the user interface to distinguish when an object is detected; to finish, they're sent through the serial port where it is wirelessly connected. The algorithm shown indicates the process performed in the first iteration, the continuous operation, would be repetitive from the data acquisition of the sensors.

## 3. Results

**Figure 7:**
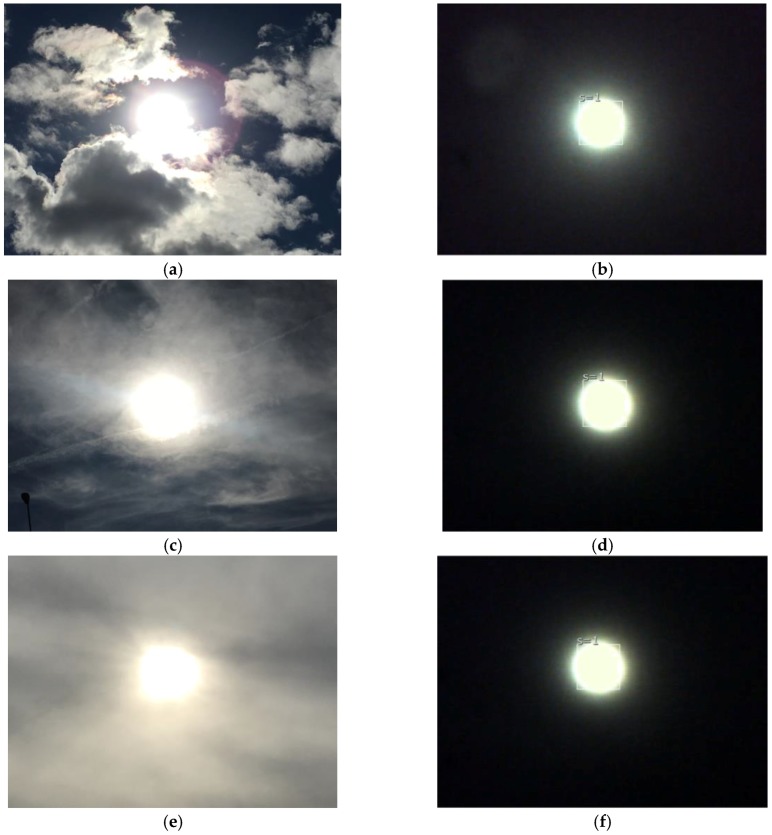
Cloudy real images and images viewed through the vision sensor. (**a**) Real view of the Sun partially cloudy; (**b**) Sensor view of the Sun partially cloudy; (**c**) Real view of the Sun nebulous cloudy; (**d**) Sensor view of the Sun nebulous cloudy; (**e**) Real view of the Sun cloudy; (**f**) Sensor view of the Sun cloudy.

In order to characterize and know the degree of error of the position sensor, tests were performed under different conditions to record its behavior and if this was affected. One of the most common events occurs when the solar position the sensor faces is clouded, Figure 7 illustrates the actual image and the view from the sensor in three different cloudy cases. In Figure 7a the Sun is partially seen, blocked by a cloud that causes the Sun’s rays to disperse, Figure 7b shows how the filter attenuates the low intensity radiation that forms the diffuse radiation aureole, allowing the view from the vision sensor to be clear, that allows an identification of the object without disturbances.

**Figure 8:**
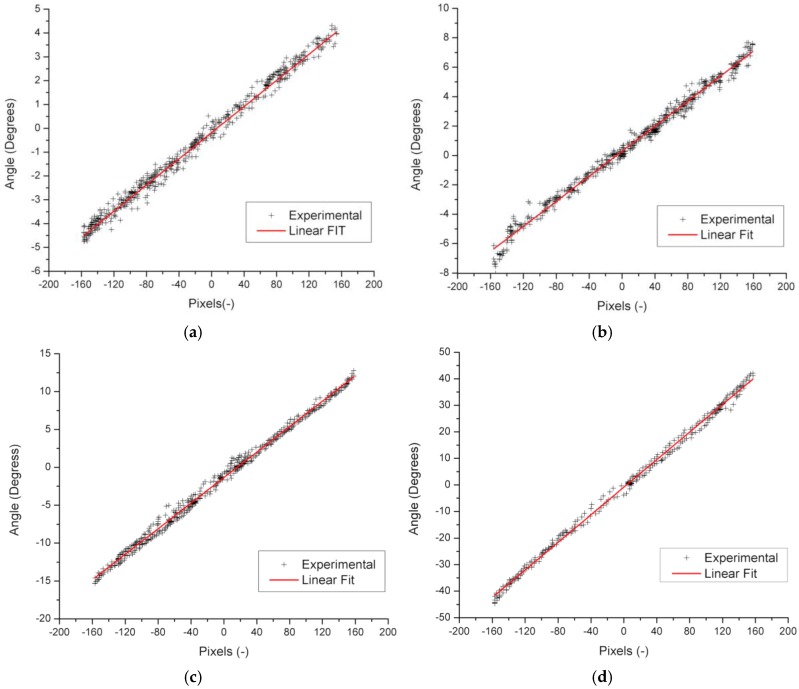
Characterization and adjustment of pixels to degrees with different lenses in degrees: (**a**) With lens of 12°; (**b**) With lens of 21°; (**c**) With lens of 42°; (**d**) With lens of 75°.

Figure 7c illustrates a complete low-intensity nebulous cloudy day, the Sun is distorted by the diffuse radiation of the clouds, and even a change in its shape is seen due to the non-homogeneity of the clouds, yet despite this, the filter manages to block the imperfections caused by the clouds (see Figure 7d), so that the sensor makes an identification of the object without disturbances. The last case of Figure 7e illustrates that the circumference of the Sun is affected by the intense overcast, but despite this, in the next shot (Figure 7f) we can see that the image from the image sensor is again clear, as if it were not cloudy. With these tests we can verify that the filter performs a perfect job at attenuating perturbations presented in this type of application [29]. The type of lens plays an important role in the capture of the image in the vision sensors, as demonstrated in the work of Lee et al. [24] which showed that the aperture of the lens helps improve the accuracy of the detection of the Sun. In this sense the Pixy vision sensor was tested with different types of lenses, choosing the four types of M12 lenses with the most common different vision angles, namely 12° (focal length 25 mm, F2.0), 21° (focal length 16 mm, F2.0), 42° (focal length 8.0 mm, F2.0) and 75° (focal length 2.8 mm, F2.0). The pixel/degree equivalence conversion factor was characterized. For this, the sensor was exposed directly to the Sun and displacements were performed on the “x” (azimuth angle) and “y” (zenith angle) axis, the corresponding angle measurements were recorded for each defocused pixel. Figure 8 shows the relation of pixels to degrees with a 12° (Figure 8a), 21° (Figure 8b), 42° (Figure 8c), and 75° (Figure 8d) lens. The data shows a linear behavior with some points of dispersion, which happens because the angle measurement is done with an inertial sensor and in a sudden movement a change in the acceleration occurs, which is corrected once the sensor stabilizes.

Table 1 illustrates the first-order adjustment equations and the accuracy for each lens. It is observed that the detection of the Sun increases as the viewing angle decreases, however, the accuracy decreases because the resolution of the inertial sensor is reaching its limit, making it more difficult to read small changes. The selection of the lens depends on the precision and accuracy required for each application, and because of this, the lens with a 21-degree viewing angle was selected, since it is a midpoint between accuracy and precision, with a 0.04260° of resolution and a setting of 99.014%.

In the adjustment equations of Table 1 the intercept for all cases is different from zero, because at the beginning of the experiment the position of the Sun did not match the zero pixel, since it is a scenario that cannot be controlled.

**Figure 9:**
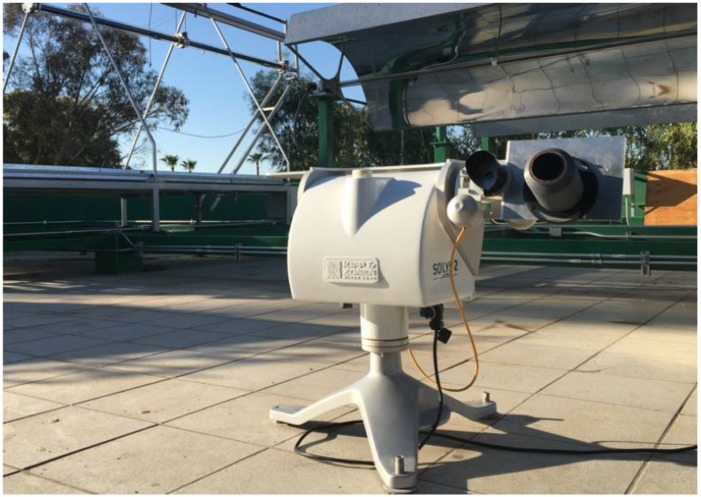
Experimental setup, SOLYS 2 and on its surface the solar position sensor.

However, it is known that it is a constant displacement and to obtain the experimental resolution (Rex), the interception can equal zero or it can have obtained by a difference as indicated by Equation (1):

(1) Rex=angle(n+1)− angle(n),

The validation of the solar position sensor was performed by determining the focus error of one of the best commercial solar tracking systems of Kipp & Zonen (SOLYS 2), which ensures a solar tracking with 0.02° of accuracy in active mode. Figure 9 shows the configuration of the experiment. The white mechanism is the SOLYS 2 with its sensor and pyreliometer. On the right side a base to place the solar position sensor can be seen. The SOLYS 2 solar tracker works independently of the solar position sensor. In the experimental study, the variables of azimuthal and zenith approach, temperature inside the sensor, ambient temperature and solar radiation were recorded every 1 s.

**Figure 10:**
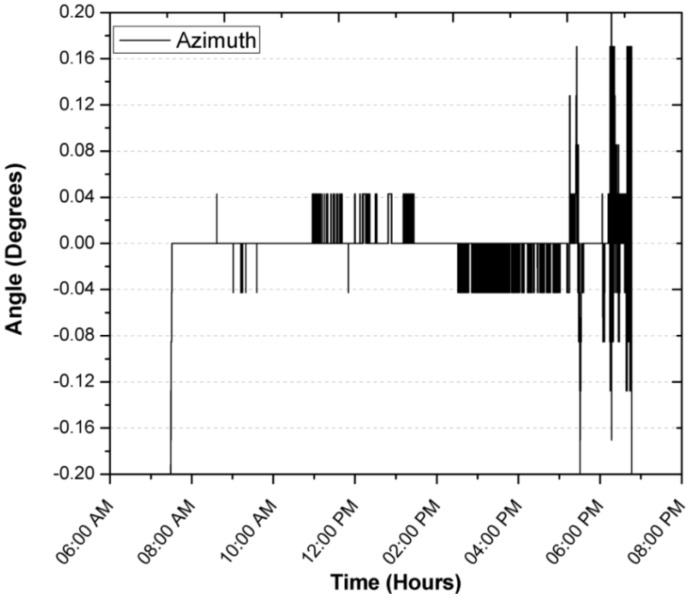
Focus error in the zenith angle during a first day of operation.

**Figure 11:**
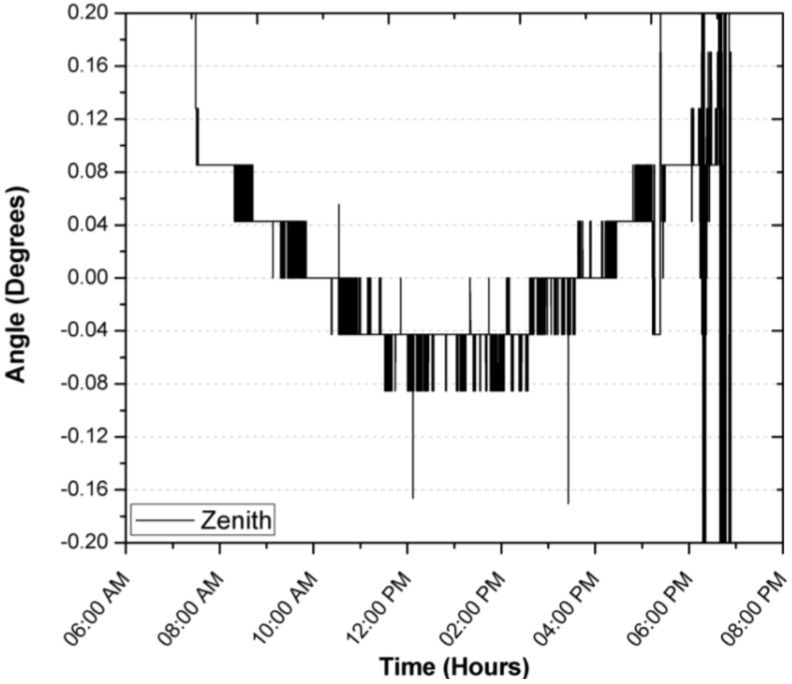
Focus error in the azimuth angle during a first day of operation.

**Figure 12:**
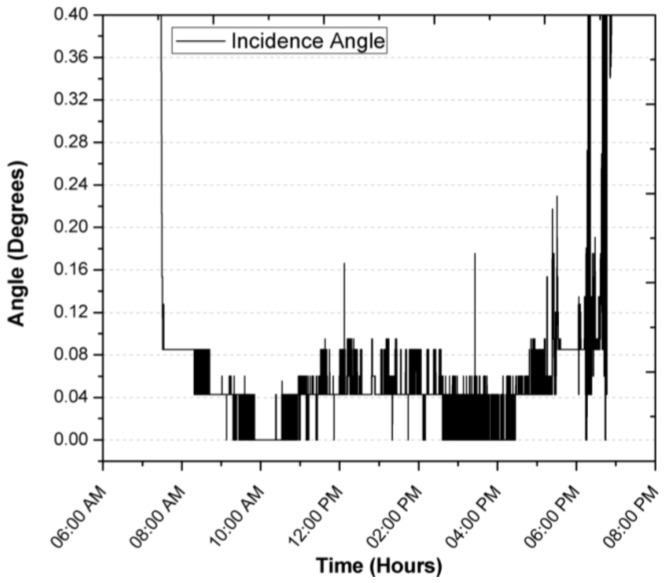
Focus error in the incidence angle during a first day of operation.

**Figure 13:**
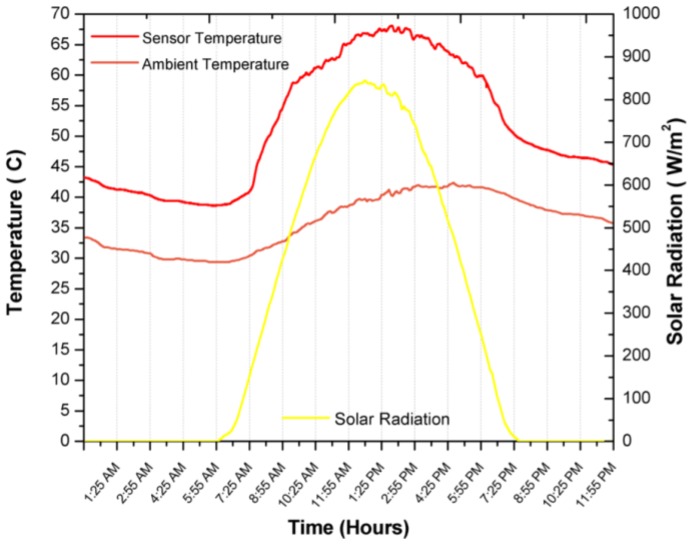
Ambient temperature and temperature inside the sensor during its operation throughout the first day.

Figure 10, Figure 11, Figure 12 and Figure 13 show the results of the experiments during a full day, where the first tree graph shows the focus error in the azimuth, zenith and incidence angle, the fourth shows the solar radiation, ambient temperature and internal temperature of the solar position sensor. Around 6:35 a.m. the sensor begins to detect the Sun with a radiation of 60 W/m^2^, the azimuth angle stays perfectly focused at 0° until around 10:00 a.m., when it presents small delays of 0.042°. It is worth mentioning that it is the first value of the discrete ramp or the resolution of the solar position sensor. The focus on the azimuth angle of the SOLYS had an excellent performance by staying within the minimum resolution of the solar position sensor, in the experiment can be seen that from 4:00 p.m. onwards, shadows can be seen in the area where the SOLYS was placed, which causes disturbances in the measurement, therefore the data was excluded and is not considered in the analysis, however, they are still shown in the graph with the purpose of presenting the experiment throughout the whole day. 

The zenith begins by detecting the Sun at the same time, maintaining an error of 0.08°, you can see the focus error changes approximately every hour, which could be translated into an error by the SOLYS solar tracker sensor or a deviation in its alignment (base, sensor or structure), despite this, its performance is very close to that one reported by the equipment manufacturer.

Figure 13 shows the solar radiation during the test period. It can be seen that the measurement of the position of the Sun is not affected by the change in solar radiation levels. Not until 4:30 p.m. when a tree is interposed between the solar position sensor and the Sun, is the circumference detected by the sensor to be different and the centroid of the object changes its position and affects its measurement, although this disturbance that is not an operation is maintained with a blur below 0.3. The overall behavior of the solar tracker can be evaluated with the result of the azimuthal and zenith angle error, called the angle of incidence, which can be visualized in Figure 12, during normal operation from 7:00 a.m. to 5:30 p.m. It is observed that the error remained below 0.044°, mainly due to the error in the zenith angle, which could have been corrected with an exact leveling or orientation, which is difficult to perform manually. On the other hand, it is seen that the change in ambient temperature does not affect the operation and performance of the solar position sensor. In Figure 13 it is illustrated that the heat generated by the devices raises the inside temperature of the sensor by an average of 9.3 °C when there is no heat input from solar radiation. During the time of solar radiation and considering the thermal inertia, it is seen that the temperature rises from 25.5 °C to 839 W/m^2^ two different things so the following makes no sense, resulting in the temperature of the interior of the solar position sensor increasing by 1 °C for every 28.91 W/m^2^, so with these data, there is an maximum estimated working operation temperature of 47 °C.

**Figure 14:**
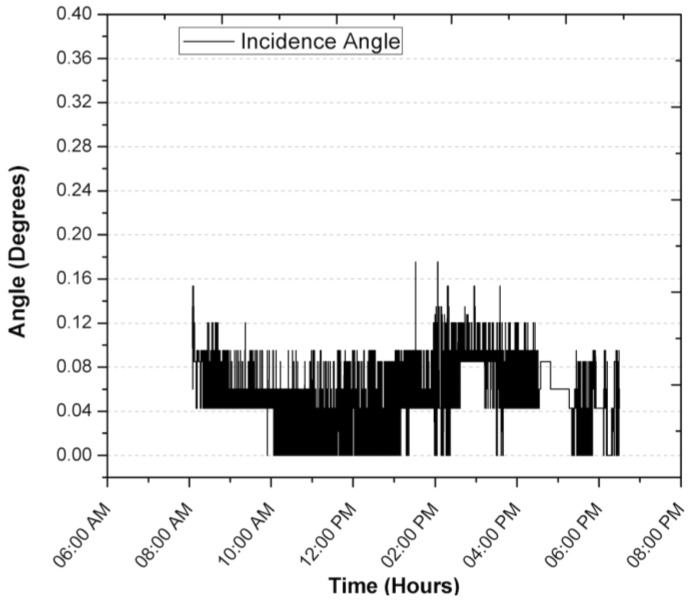
Focus error in the incidence angle during a second day of operation.

**Figure 15:**
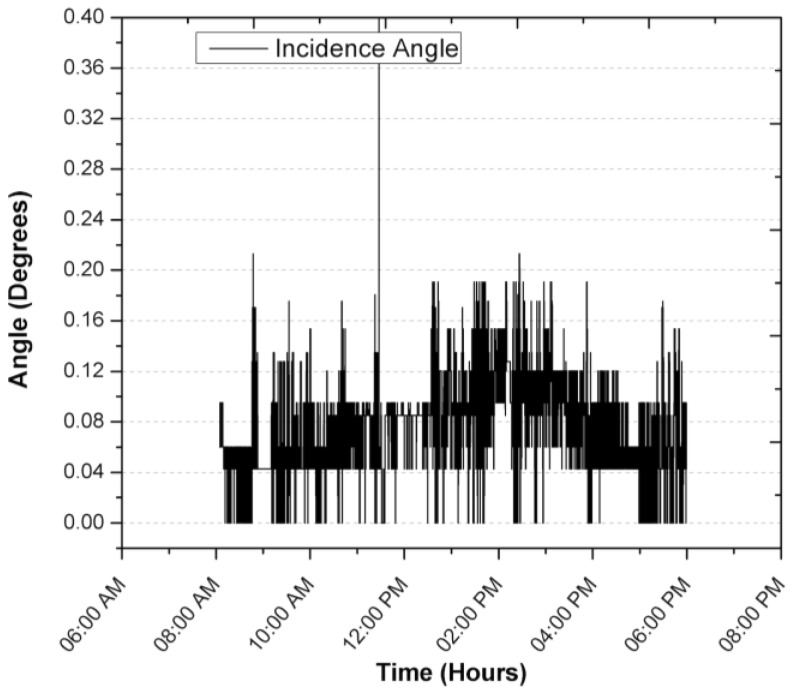
Focus error in the incidence angle during a third day of operation.

During the registration of the first day of operation of the sensor, there was an average focus error of 0.0065°, 0.04196° and 0.04588° for the azimuth, zenith and incidence angles, with modes of 0°, 0.0426° and 0.0426°, resulting in the largest error being obtained for the angle zenith. After explaining a day of operation in detail, and in order to demonstrate the consistency of the sensor, the error of the angle of incidence in two days of operation is shown in Figure 14 and Figure 15. The incidence angle error of the second day indicates 0.02039°, 0.04021° and 0.04304° for the azimuth, zenith and incidence angles, with modes of 0°, 0.0426° and 0.0426°. On the third day we obtained 0.04885°, 0.04172° and 0.07455° for the azimuth, zenith and incidence angles, with a mode of 0.0426°, 0.0426° and 0.0426°.

With this it is possible to validate a sensor precision of between 0° and 0.0426° for the azimuth, zenith and incidence angles, just a step in the overall resolution of the sensor as indicated in Table 1 for the lens with 21° of vision angle. The resolution of 0.239° per pixel and a viewing angle of 75° is enough for solar tracking systems applied in photovoltaic systems or in some solar collectors, such as a parabolic cylinder which efficiency starts to decrease at 0.5° [9]. According to the results it is proposed that for applications that require greater accuracy, such as the evaluation of tracking systems, the lens should be exchanged for ones with a lower viewing angle, or with magnifying lenses where resolutions up to 0.0017° have been reached for each pixel [24].

## 4. Conclusions

With the development of a solar position sensor based on a vision system and the tests performed, it is concluded that a measurement of the error of approach or position of the Sun can be made from a reference, with accuracies of 0.0258° to 0.2396° and viewing angles from 12 to 75 with an uncertainty of 0.2 to 1.55%, which can be modified to achieve accuracies of less than 0.01°. The vision system proved to be a very good choice for processing and determining the position of the sun, without its accuracy being affected by changes in ambient temperature or solar radiation levels. In addition to the above it is recommended to use different lenses to find the right balance between the accuracy and angle of vision of the application.

The darkened glass used and the tests demonstrate that it is an excellent technique to attenuate disturbances by refraction or dispersion of sunlight, helping to obtain better measurements of the location of the Sun, in addition to filtering excessive light to the vision sensor, decreasing the temperature and unnecessary wavelengths, resulting in an increase in sensor life.

The solar position sensor based on a vision sensor gave the expected behavior, maintaining a precision in the computation of the focus error of the angles azimuth, zenith and incidence of 0 to 0.0426, therefore the proposed sensor complies with the requirements for detection of the Sun and components that support adequate environmental conditions and accuracy requirements to be used like a sensor to obtain the focus error and thereby evaluate solar tracking systems, in addition like a sensor to feedback solar tracking systems or on orientation devices, leveling and positioning in the installation of photovoltaic plants and solar collectors.

## Figures and Tables

**Table 1:** Accuracy and coefficients of the first order adjustment equation for the different types of lenses.

Vision Angle	Slope	Interception	Adjustment
12	0.02583	0.24189	98.448%
21	0.04260	0.28362	99.014%
40	0.08420	−1.35669	99.518%
75	0.23962	−0.84907	99.721%
